# Supplementary material for: Auditory closed-loop stimulation of EEG slow oscillations strengthens sleep and signs of its immune-supportive function
Source: Nat Commun. 2017 Dec 7;8:1984. doi: 10.1038/s41467-017-02170-3 (PMC5719447; doi:10.1038/s41467-017-02170-3)
Supplement: Supplementary file 1 — Supplementary Information [file 41467_2017_2170_MOESM1_ESM.pdf]

Supplementary Figures

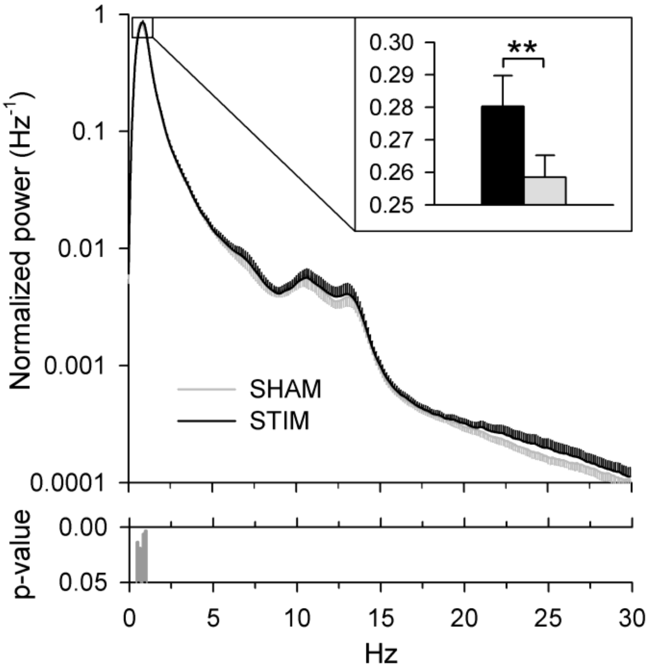

Supplementary Figure 1. Auditory closed-loop stimulation selectively enhances slow oscillatory activity. Mean (± s.e.m.) normalized spectral power during stimulation for electrode position Fz determined for NonREM epochs of the 120-min stimulation period for the Stimulation (STIM, black) and Sham condition (SHAM, grey) for frequencies up to 30 Hz. For normalization, individual spectra were divided by the cumulative power (up to 30 Hz). The bottom panel indicates significance between the effects of the Stimulation and Sham condition (paired t-tests, two-sided). The insert shows the normalized power in the SO peak frequency band (0.8-1.1 Hz); **$p < 0.01$. n = 14.

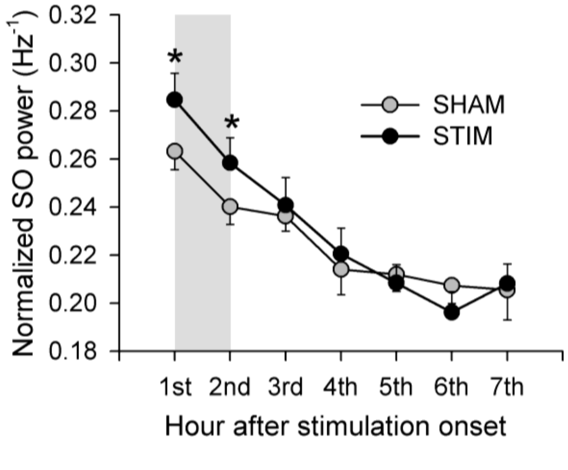

Supplementary Figure 2. Time course of slow oscillatory activity across the whole night. Mean (± s.e.m.) normalized slow oscillatory (SO) activity across the whole night in one-hour intervals starting with stimulation onset. For normalization, individual spectra were divided by the cumulative power (up to 30 Hz). *$p < 0.05$ for pairwise comparisons between the Stimulation condition (STIM, black) and the Sham condition (SHAM, grey) with paired t-tests, two-sided. n = 14. Please notice that no SO activity could be calculated for the 8th hour as most subjects did not display any NonREM sleep at that time.

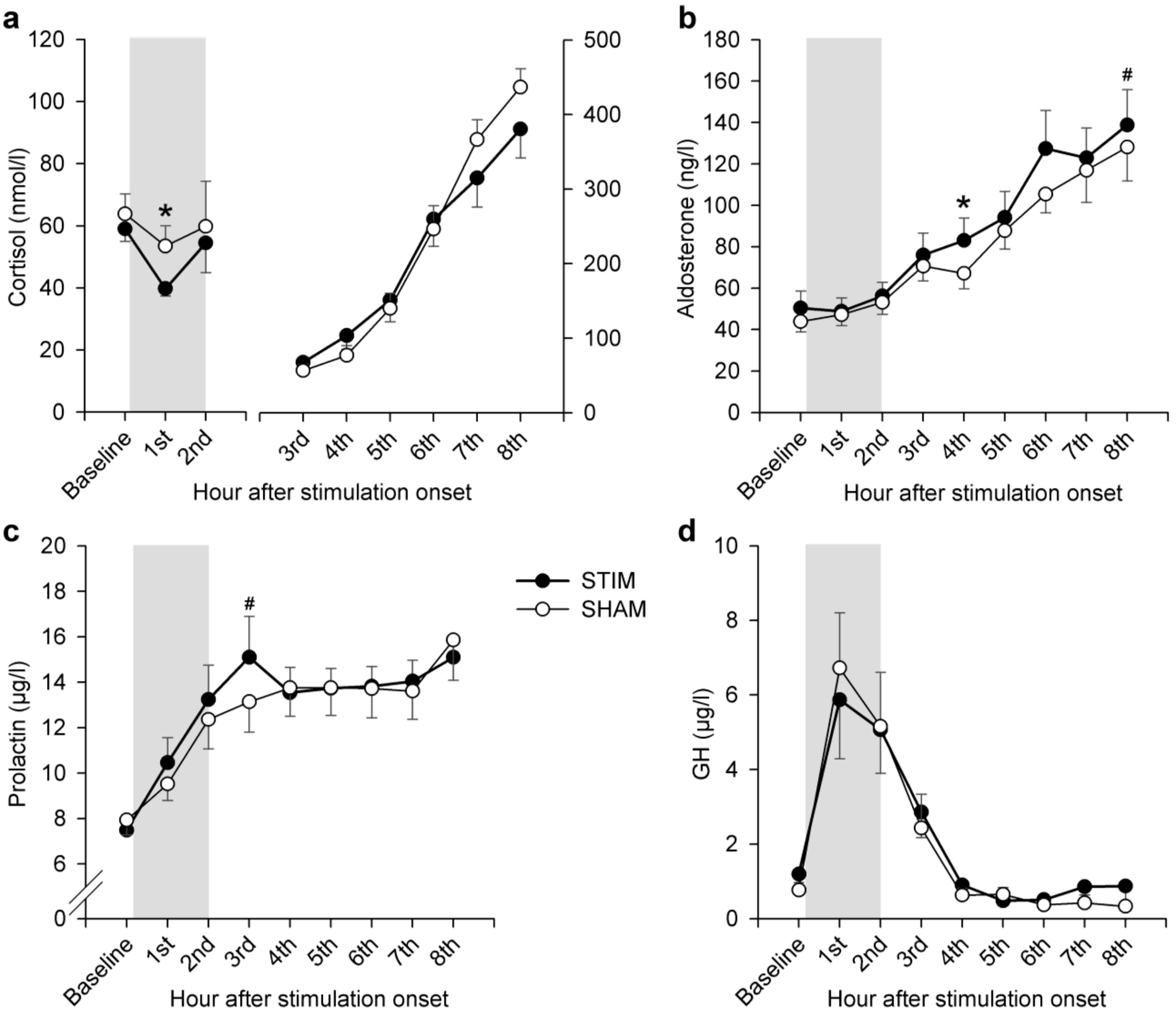

Supplementary Figure 3. Time course of cortisol, aldosterone, prolactin, and growth hormone levels with and without auditory SO stimulation. Means (± s.e.m.) of cortisol (a), aldosterone (b), prolactin (c), and growth hormone (GH) (d) levels calculated for one-hour bins starting from stimulation onset. Grey area represents the 120-min stimulation period. *$p < 0.05$, $^{\#}p < 0.1$ for pairwise comparisons between the Stimulation condition (STIM, black) and the Sham condition (SHAM, white) with Wilcoxon tests, two-sided. n = 10–14 (see "Methods" section for exact numbers). Please note in (a) the different scaling of the y-axis for cortisol levels during and post stimulation, respectively, which was applied because of the strong circadian rhythm of this hormone.
